# Bone-Targeted Therapies and Spinal Morphometry in Postmenopausal Osteoporosis: A Real-World Longitudinal Retrospective Study

**DOI:** 10.3390/bioengineering13070800

**Published:** 2026-07-13

**Authors:** Zhen-Wei Wu, Szu-Wei Chen, Sz-Huei Li, Wen-Tien Wu, Cheng-Huan Peng, Hao-Wen Chen, Chia-Ming Chang, Tzai-Chiu Yu, Ing-Ho Chen, Jen-Hung Wang, Kuang-Ting Yeh

**Affiliations:** 1 Department of Medical Education, Taipei Tzu Chi Hospital, Buddhist Tzu Chi Medical Foundation, Taipei 231405, Taiwan; 108311155@gms.tcu.edu.tw (Z.-W.W.);; 2 School of Medicine, Tzu Chi University, Hualien 970374, Taiwan; 3 Department of Orthopedics, Hualien Tzu Chi Hospital, Buddhist Tzu Chi Medical Foundation, Hualien 970473, Taiwan; 4 Institute of Medical Sciences, Tzu Chi University, Hualien 970374, Taiwan; 5 Department of Medical Research, Hualien Tzu Chi Hospital, Buddhist Tzu Chi Medical Foundation, Hualien 970473, Taiwan; paulwang@tzuchi.com.tw; 6 Department of Medical Education, Hualien Tzu Chi Hospital, Buddhist Tzu Chi Medical Foundation, Hualien 970473, Taiwan; 7 Graduate Institute of Clinical Pharmacy, Tzu Chi University, Hualien 970374, Taiwan

**Keywords:** osteoporosis, postmenopausal, bone density conservation agents, anabolic agents, spinal curvatures, lumbar vertebrae

## Abstract

This 24-month retrospective, exploratory observational study evaluated longitudinal spinal morphometric changes in 128 postmenopausal women with osteoporosis receiving anabolic therapy (*n* = 30) or non-anabolic anti-osteoporotic therapy (*n* = 98). Lumbar BMD, lower lumbar vertebral heights, disc height indices, and sagittal spinopelvic parameters were assessed at baseline and 24 months. Unweighted and baseline-severity adjusted linear regression models were used; IPTW and overlap-weighted analyses were performed as sensitivity analyses. Both groups showed significant lumbar BMD improvement. Patients receiving anabolic therapy were older and had lower baseline lumbar BMD and greater thoracolumbar kyphosis, indicating confounding by indication. In unweighted models, anabolic therapy was associated with greater L5 anterior height loss and L5/S1 disc height index preservation; however, these associations were attenuated after adjustment for baseline lumbar BMD and thoracolumbar kyphosis and were not consistently supported by propensity-score-weighted sensitivity analyses. Pelvic incidence showed more consistent level-specific associations, being negatively associated with L5 anterior height change and positively associated with L5/S1 disc height index preservation. Given substantial confounding by indication and limited treatment-group overlap, treatment-related associations should not be interpreted causally. These findings suggest that spinopelvic morphology may influence lower lumbar structural adaptation, whereas treatment-related morphometric findings remain exploratory and require confirmation.

## 1. Introduction

Osteoporosis is a systemic skeletal disorder characterized by low bone mass and microarchitectural deterioration, leading to increased bone fragility and susceptibility to fractures [1]. It affects an estimated 200 million people worldwide, and its prevalence increases with age, particularly among postmenopausal women. Vertebral compression fractures are the most common osteoporotic fractures, occurring in approximately 25% of postmenopausal women; nevertheless, up to two-thirds of these fractures remain clinically unrecognized [2]. Beyond the acute morbidity associated with fracture events, vertebral fractures have profound long-term consequences, including chronic back pain, progressive spinal deformity, height loss, restrictive pulmonary dysfunction, and significantly impaired quality of life [3]. Kado et al. demonstrated that each vertebral fracture increased mortality risk by 23%, with a dose-dependent effect related to the number and severity of fractures [4]. Furthermore, patients with prevalent vertebral fractures face a five-fold increased risk of subsequent vertebral fractures and a two- to three-fold increased risk of hip and other nonvertebral fractures, creating a cascade of progressive skeletal fragility. The economic burden is substantial, with osteoporotic fractures accounting for over $19 billion in annual healthcare costs in the United States alone [5]. These observations underscore the importance of effective pharmacological interventions to prevent fractures, preserve bone structure, and maintain spinal alignment in patients with osteoporosis.

The current pharmacological management of osteoporosis encompasses two major therapeutic approaches: antiresorptive agents and anabolic agents, which differ fundamentally in their mechanisms of action and effects on bone remodeling. Antiresorptive agents, including bisphosphonates and denosumab, inhibit osteoclast-mediated bone resorption, thereby reducing bone turnover and allowing continued bone formation to gradually increase bone mass. These agents have demonstrated consistent efficacy in reducing vertebral fracture risk by 40–70% in large randomized controlled trials and have been the cornerstone of osteoporosis treatment for decades [6]. Conversely, anabolic agents, including teriparatide and romosozumab, stimulate bone formation by activating osteoblasts and increasing bone remodeling, with a net positive bone balance. Clinical trials have demonstrated that anabolic agents produce more rapid and substantial increases in bone mineral density than antiresorptive agents, with reductions in vertebral fracture risk of 65–86% observed in pivotal trials [7]. Current treatment guidelines from the American Association of Clinical Endocrinologists and the Endocrine Society recommend anabolic agents as first-line therapy for patients at extremely high fracture risk, defined as those with T-scores ≤ −3.0, multiple prevalent fractures, recent fractures, or fractures occurring while on osteoporosis therapy [8]. Although the efficacies of both anabolic and antiresorptive agents in increasing bone mineral density and reducing fracture risk have been well established in randomized controlled trials, their comparative effects on vertebral morphology and spinal alignment in real-world clinical settings remain incompletely characterized [9]. Most clinical trials have focused on dual-energy X-ray absorptiometry (DXA)-measured bone mineral density (BMD) changes and radiographically confirmed fracture incidence as primary endpoints, with limited attention to detailed morphometric changes in vertebral body dimensions, disc space heights, and sagittal spinal alignment parameters [10]. Nonetheless, these structural parameters may be clinically relevant as structural surrogate markers because progressive vertebral height loss and spinal deformities, even in the absence of acute fracture events, contribute significantly to functional impairment, chronic pain, and reduced quality of life in patients with osteoporosis. Furthermore, the relationship between BMD improvement and structural preservation may not be linear or uniform across different vertebral levels, as regional variations in trabecular density, cortical thickness, and biomechanical loading patterns may influence the treatment response. Recent biomechanical studies using finite element analysis suggested that anabolic agents may improve bone quality and vertebral strength beyond those predicted by BMD changes alone, potentially through improvements in trabecular microarchitecture, cortical thickness, and endplate integrity [11]. However, direct comparative evidence for differential effects of anabolic versus antiresorptive therapy on vertebral morphometric parameters in clinical populations remains limited, particularly in patients with established spinal deformities, who represent a high-risk population frequently encountered in clinical practice.

Recent cross-sectional studies have identified several key factors associated with spinal health and functional outcomes in patients with osteoporosis. Global Tilt (GT) has emerged as a critical indicator of spinal malalignment, with higher GT values correlating with reduced functional independence and increased disability [12]. Grip strength is a valuable functional marker, and greater grip strength is associated with better spinal alignment and higher functional scores [13]. In patients with vertebral fractures, a thoracolumbar kyphotic angle > 14° has been identified as a threshold predictive of severe pain and disability, potentially indicating the need for intervention [14]. Furthermore, aging necessitates compensatory spinal changes, such as increased cervical lordosis, to maintain sagittal balance [15]. Nevertheless, these cross-sectional observations could not establish temporal relationships or causality between vertebral deformation, disc degeneration, and functional decline. Longitudinal studies are therefore essential to understand the progressive nature of osteoporotic spinal changes and to identify modifiable risk factors. Despite extensive research on osteoporotic vertebral fractures, the longitudinal relationship between vertebral height changes and adjacent disc degeneration remains poorly understood. Although previous studies have documented the natural history of vertebral compression fractures [4,16], few have systematically evaluated the temporal relationship between gradual vertebral body deformation and disc space narrowing using standardized morphometric methods. Furthermore, the roles of spinopelvic alignment and bone mineral density in modulating these structural changes have not been adequately examined.

Therefore, this retrospective observational study aimed to evaluate longitudinal changes in vertebral morphometric parameters and adjacent disc height indices in postmenopausal women with osteoporosis over 2 years. The specific objectives were to: (1) quantify changes in vertebral body heights at L4 and L5; (2) assess changes in disc height indices at L4–L5 and L5–S1 using Farfan’s method; (3) examine the relationship between vertebral height loss and adjacent disc degeneration; (4) identify independent predictors of morphometric changes, including bone mineral density and spinopelvic parameters; and (5) evaluate concurrent changes in sagittal spinal alignment. We hypothesized that progressive vertebral height loss would be associated with accelerated adjacent disc height decline, modulated by baseline bone quality and spinopelvic alignment. These findings may help elucidate the biomechanical cascade of osteoporotic spinal degeneration and inform clinical monitoring strategies.

## 2. Materials and Methods

### 2.1. Study Design and Participants

This retrospective, longitudinal, exploratory observational cohort study was conducted at the Department of Orthopedic Surgery, Hualien Tzu Chi Hospital, Buddhist Tzu Chi Medical Foundation, a tertiary referral center in eastern Taiwan. The study protocol was approved by the Research Ethics Committee of Hualien Tzu Chi Hospital (IRB no. IRB113–208-B) and was conducted in accordance with the Declaration of Helsinki and the Strengthening the Reporting of Observational Studies in Epidemiology (STROBE) recommendations. Medical records were reviewed for postmenopausal women managed between 1 January 2020, and 30 June 2023, with radiographic and densitometric follow-up extending to 24 months.

Postmenopausal women aged 50 years or older were eligible if they had osteoporosis diagnosed by dual-energy X-ray absorptiometry (DXA; Explorer, Hologic, Waltham, MA, USA), defined as a T-score ≤ −2.5 at the lumbar spine, femoral neck, or total hip according to World Health Organization criteria. Because the objective of the study was to examine longitudinal spinal morphometric change in osteoporotic patients with radiographic spinal involvement, eligible participants were required to have available standing spinal or lumbar radiographs at baseline and at the 24-month follow-up. Evidence of spinal involvement was defined as thoracolumbar kyphosis or vertebral deformity identified on standing lateral radiographs. Radiographic examinations and DXA measurements were performed as part of routine clinical care.

Patients were excluded if they had a history of spinal fusion or instrumentation before the baseline assessment; prior major lower limb arthroplasty; traumatic pelvic or lower limb fracture during the follow-up period; acute vertebral compression fracture during the study period; inability to maintain an upright standing posture for radiographic assessment; inability to ambulate independently without a walker or walking aid for at least 50 m; secondary osteoporosis caused by endocrine, renal, metabolic, or medication-related conditions; metabolic bone disease other than osteoporosis; severe spinal deformity precluding reliable measurement, including scoliosis with Cobb angle >20°; or incomplete baseline or follow-up radiographic data for the primary morphometric outcomes. After applying the eligibility criteria, 128 postmenopausal women were included in the final analysis, comprising 30 patients who received anabolic therapy and 98 patients who received non-anabolic anti-osteoporotic therapy.

### 2.2. Treatment Exposure

The treatment exposure of interest was use of anabolic anti-osteoporotic therapy during the observation period. Patients were categorized into two groups according to whether they received anabolic therapy. The anabolic group included patients treated with teriparatide or romosozumab, whereas the non-anabolic group included patients treated with antiresorptive or non-anabolic agents, including zoledronic acid, denosumab, and raloxifene. Treatment allocation was determined by clinical judgment in routine practice rather than randomization. Therefore, the treatment comparison was interpreted as an observational association and not as a causal treatment effect. Because patients receiving anabolic therapy were expected to have greater baseline disease severity, confounding by indication was specifically addressed using baseline-severity adjusted regression and propensity-score-weighted sensitivity analyses. Because teriparatide, romosozumab, zoledronic acid, denosumab, and raloxifene have distinct mechanisms of action, the treatment categories should be interpreted as pragmatic real-world exposure groups rather than biologically interchangeable drug classes. The sample size did not permit reliable agent-specific comparative analyses.

### 2.3. Bone Mineral Density Assessment

Bone mineral density was measured at baseline and at 24 months using DXA (Explorer, Hologic, Waltham, MA, USA). Lumbar spine BMD was calculated from L1–L4 according to standard densitometric protocols. Individual L4 BMD was also recorded because the primary morphometric analyses focused on the lower lumbar spine. Change in BMD was calculated as the 24-month value minus the baseline value. Positive values therefore indicated BMD gain during follow-up. Longitudinal BMD analyses in this study were restricted to lumbar spine measurements because femoral neck and total hip BMD were not systematically available for all participants across both time points.

### 2.4. Radiographic Morphometric Measurements

Standing lateral radiographs of the thoracolumbar or lumbar spine were obtained at baseline and at 24 months using standardized clinical protocols. Patients were positioned in a standing lateral posture with the arms supported or crossed to reduce superimposition over the spine. Radiographs were assessed using a picture archiving and communication system workstation. Two trained observers independently performed radiographic measurements using a standardized measurement protocol and were blinded to clinical treatment data and the temporal sequence of radiographs. When discrepancies greater than 5% occurred, consensus was reached through discussion with a senior spine surgeon. Measurement reliability was evaluated in a randomly selected reliability subsample, in which radiographs were remeasured after a 4-week interval. Intraclass correlation coefficients greater than 0.80 were considered to indicate good-to-excellent reliability.

Anterior and posterior vertebral body heights were measured at L4 and L5. Anterior height was defined as the distance between the superior and inferior endplates along the anterior vertebral wall, whereas posterior height was defined as the corresponding distance along the posterior vertebral wall. Vertebral width measurements were also obtained where applicable. Changes in vertebral height or width were calculated as the 24-month value minus the baseline value; negative values indicated height loss or width reduction.

Intervertebral disc height was assessed at L4/5 and L5/S1. Anterior and posterior disc heights were measured between the adjacent vertebral endplates. Disc height index was calculated using Farfan’s method to account for interindividual differences in vertebral dimensions and radiographic magnification. Changes in disc height index were calculated as the 24-month value minus the baseline value, with negative values indicating disc height index loss.

Sagittal alignment parameters included pelvic incidence (PI), thoracolumbar kyphosis (TLK), upper lumbar lordosis (ULL), lower lumbar lordosis (LLL), and sacral slope (SS). TLK was measured between the superior endplate of T10 and the inferior endplate of L2. ULL and LLL were measured using standard Cobb-based methods. PI and SS were measured using established spinopelvic definitions. Kyphotic angles were recorded as positive values, and lordotic angles were recorded according to the institutional measurement convention used in the dataset.

### 2.5. Outcomes

The principal outcomes were 24-month changes in lower lumbar vertebral morphometry and disc height index. Four outcomes were selected for multivariable modeling based on clinical relevance and their focus on lower lumbar vertebral and disc morphometry: change in L4 anterior vertebral height, change in L5 anterior vertebral height, change in L4/5 disc height index, and change in L5/S1 disc height index. The main exposure variable was anabolic therapy status. The primary analytic objective was to determine whether anabolic therapy status was associated with these morphometric outcomes after adjustment for baseline disease severity.

### 2.6. Statistical Analysis

Continuous variables were summarized as mean ± standard deviation, and categorical variables were summarized as number and percentage. Between-group comparisons at baseline were performed using Welch’s *t*-test for continuous variables because of unequal group sizes and potential variance heterogeneity. Categorical medication distributions were compared using chi-square testing. Standardized mean differences were calculated to quantify baseline imbalance between treatment groups; an absolute standardized mean difference greater than 0.10 was considered indicative of imbalance. Within-group changes from baseline to 24 months were analyzed using paired *t*-tests. Between-group differences in change values were analyzed using Welch’s *t*-test. For variables with incomplete follow-up measurements, available-case analyses were performed, and variable-specific sample sizes were reported in the tables. All change values were calculated as follow-up minus baseline; therefore, negative coefficients or mean differences indicated structural loss or reduction over time. Multivariable linear regression models were constructed for the four primary morphometric outcomes. The unweighted model included age, anabolic therapy status, PI, and change in lumbar BMD. To address potential confounding by indication related to baseline disease severity, a second baseline-severity adjusted model additionally included baseline lumbar BMD and baseline TLK. This model was considered the principal confounding-adjusted model for manuscript interpretation. Regression coefficients were reported with 95% confidence intervals, nominal *p*-values, FDR-adjusted q-values, adjusted R^2^, sample size, and sample-to-predictor ratio. Given the modest size of the anabolic subgroup, no anabolic-only multivariable regression model was performed. As sensitivity analyses, propensity-score-weighted models were performed. The propensity score was estimated using logistic regression with anabolic therapy status as the dependent variable and age, PI, baseline lumbar BMD, and baseline TLK as covariates. Stabilized inverse probability of treatment weighting (IPTW) and overlap weighting were then applied. Balance before and after weighting was assessed using weighted means and standardized mean differences. Weighted regression models were estimated using robust standard errors. Because stabilized IPTW generated extreme weights and a low effective sample size in the control group, IPTW and overlap-weighted models were interpreted as supplementary sensitivity analyses rather than primary causal estimates. To account for the exploratory nature of multiple regression testing, Benjamini–Hochberg false discovery rate correction was applied across the non-intercept coefficients in the primary models and supplementary weighted models. Statistical significance was defined by a two-sided nominal *p*-value <0.05; however, findings with nominal significance but nonsignificant FDR-adjusted q-values were interpreted cautiously as hypothesis-generating.

## 3. Results

### 3.1. Baseline Characteristics

A total of 128 postmenopausal women with osteoporosis were included in the analytic cohort. Thirty patients received anabolic therapy, and 98 patients received non-anabolic anti-osteoporotic therapy. The anabolic group consisted of 25 patients treated with teriparatide and 5 treated with romosozumab, whereas the non-anabolic group consisted of patients treated with zoledronic acid, denosumab, or raloxifene. The medication distribution differed significantly between groups, as expected from the exposure definition (*p* < 0.001) (Table 1). Substantial baseline imbalance was observed between the two treatment groups. Patients receiving anabolic therapy were significantly older than those receiving non-anabolic therapy (70.53 ± 4.26 vs. 66.93 ± 6.73 years, *p* < 0.001; standardized mean difference [SMD] = 0.58). Baseline lumbar BMD was markedly lower in the anabolic group than in the non-anabolic group (0.763 ± 0.126 vs. 0.929 ± 0.104 g/cm^2^, *p* < 0.001; SMD = −1.51). Similarly, L4 BMD was lower in the anabolic group (0.792 ± 0.126 vs. 1.034 ± 0.142 g/cm^2^, *p* < 0.001; SMD = −1.75). These findings indicate that patients receiving anabolic therapy had substantially more severe baseline osteoporosis.

Sagittal alignment also differed between groups. Baseline TLK was significantly greater in the anabolic group than in the non-anabolic group (20.02 ± 10.16° vs. 11.56 ± 9.45°, *p* < 0.001; SMD = 0.88), indicating greater baseline thoracolumbar kyphotic deformity. PI tended to be lower in the anabolic group, although this difference did not reach statistical significance (45.88 ± 13.74° vs. 50.49 ± 10.69°, *p* = 0.099; SMD = −0.40). ULL, LLL, and SS did not differ significantly between groups. Several baseline lower lumbar morphometric and disc measurements also differed between groups. L4 anterior vertebral height was higher in the anabolic group than in the non-anabolic group (29.44 ± 2.44 vs. 28.37 ± 2.53 mm, *p* = 0.043), whereas L5 anterior height showed only a nonsignificant trend toward a higher value in the anabolic group (29.12 ± 1.97 vs. 28.24 ± 3.80 mm, *p* = 0.094). The anabolic group had greater L5 upper vertebral width (37.23 ± 2.50 vs. 35.35 ± 3.70 mm, *p* = 0.002). Disc-related measurements were also higher in the anabolic group, including L4/5 anterior disc height (13.29 ± 3.46 vs. 11.02 ± 3.24 mm, *p* = 0.003), L4/5 posterior disc height (8.63 ± 1.78 vs. 7.34 ± 2.56 mm, *p* = 0.003), L5/S1 anterior disc height (14.69 ± 6.52 vs. 11.89 ± 4.50 mm, *p* = 0.035), L4/5 height index (30.13 ± 7.06 vs. 26.08 ± 6.98, *p* = 0.008), and L5/S1 height index (33.93 ± 12.67 vs. 28.16 ± 9.77, *p* = 0.027). Collectively, the baseline data demonstrated clinically important imbalance between groups, particularly with respect to age, lumbar BMD, L4 BMD, and TLK.

### 3.2. Changes in BMD, Sagittal Alignment, Vertebral Morphometry, and Disc Height Index

Both treatment groups demonstrated significant 24-month improvement in lumbar BMD (Table 2). In the non-anabolic group, lumbar BMD increased from 0.929 ± 0.104 to 0.960 ± 0.110 g/cm^2^, with a mean change of 0.031 ± 0.070 g/cm^2^ (*p* < 0.001). In the anabolic group, lumbar BMD increased from 0.763 ± 0.126 to 0.826 ± 0.069 g/cm^2^, with a mean change of 0.062 ± 0.078 g/cm^2^ (*p* < 0.001). Although the anabolic group showed a numerically greater increase in lumbar BMD, the between-group difference in BMD change did not reach conventional statistical significance (*p* = 0.055). Similar findings were observed for L4 BMD, which increased significantly within both groups. The mean L4 BMD change was 0.048 ± 0.072 g/cm^2^ in the non-anabolic group and 0.086 ± 0.104 g/cm^2^ in the anabolic group, with a nonsignificant between-group comparison (*p* = 0.073). Sagittal alignment parameters were largely stable over 24 months. TLK, ULL, LLL, and SS did not show significant between-group differences in change values. Although SS decreased within the anabolic group, the between-group comparison was not significant; therefore, this isolated within-group finding should be interpreted cautiously.

At the vertebral body level, L4 anterior height decreased significantly in the non-anabolic group (−0.83 ± 2.06 mm, *p* < 0.001) but not in the anabolic group (−0.60 ± 2.40 mm, *p* = 0.184), and the between-group difference was not significant (*p* = 0.634). L4 posterior height decreased in the non-anabolic group (−0.57 ± 2.43 mm, *p* = 0.022) but increased in the anabolic group (0.95 ± 1.52 mm, *p* = 0.002), resulting in a significant between-group difference (*p* < 0.001). In contrast, L5 anterior height showed the most prominent between-group difference. The non-anabolic group had a nonsignificant mean change of −0.34 ± 3.51 mm (*p* = 0.344), whereas the anabolic group demonstrated a significant reduction of −2.63 ± 3.27 mm (*p* < 0.001). The between-group difference in L5 anterior height change was significant (*p* = 0.002).

Disc height changes were more heterogeneous. L4/5 posterior disc height decreased significantly in both the non-anabolic group (−0.58 ± 1.89 mm, *p* = 0.003) and the anabolic group (−0.91 ± 1.82 mm, *p* = 0.010), but the between-group difference was not significant (*p* = 0.386). L5/S1 anterior disc height decreased significantly in the non-anabolic group (−0.62 ± 2.18 mm, *p* = 0.006) but did not change significantly in the anabolic group (0.81 ± 4.73 mm, *p* = 0.356); however, the between-group difference was not statistically significant (*p* = 0.119). L4/5 height index and L5/S1 height index did not show statistically significant between-group differences in unadjusted change comparisons, although L5/S1 height index numerically increased in the anabolic group and decreased in the non-anabolic group (2.33 ± 10.43 vs. −0.93 ± 5.37, *p* = 0.109).

### 3.3. Multivariable Predictors of Lower Lumbar Morphometric Changes

The unweighted and baseline-severity-adjusted multivariable regression models are shown in Table 3. For change in L4 anterior height, anabolic therapy was not independently associated with the outcome in either the unweighted model (β = −0.209 mm, 95% CI −1.142 to 0.724, *p* = 0.659) or the baseline-severity-adjusted model (β = 0.829 mm, 95% CI −0.264 to 1.922, *p* = 0.136). In the baseline-severity-adjusted model, change in lumbar BMD was positively associated with change in L4 anterior height (β = 10.561 mm per g/cm^2^, 95% CI 4.963 to 16.159, *p* < 0.001; FDR *q* = 0.002), and baseline lumbar BMD was also positively associated with the outcome (β = 6.645, 95% CI 2.981 to 10.308, *p* < 0.001; FDR *q* = 0.003). For change in L5 anterior height, the unweighted model showed that anabolic therapy was associated with greater L5 anterior height loss (β = −2.795 mm, 95% CI −4.228 to −1.363, *p* < 0.001; FDR *q* = 0.001). PI was also negatively associated with change in L5 anterior height (β = −0.105 mm per degree, 95% CI −0.156 to −0.053, *p* < 0.001; FDR *q* = 0.001). However, after additional adjustment for baseline lumbar BMD and baseline TLK, the association between anabolic therapy and L5 anterior height change was attenuated and no longer reached conventional statistical significance (β = −1.640 mm, 95% CI −3.368 to 0.088, *p* = 0.063; FDR *q* = 0.128). In contrast, PI remained significantly and negatively associated with L5 anterior height change after baseline-severity adjustment (β = −0.104 mm per degree, 95% CI −0.155 to −0.052, *p* < 0.001; FDR *q* = 0.001). Baseline lumbar BMD showed a nominal positive association with L5 anterior height change (β = 6.157, 95% CI 0.365 to 11.949, *p* = 0.037), although this association did not remain significant after FDR correction (*q* = 0.094).

For change in L4/5 height index, anabolic therapy was not significantly associated with the outcome in either model. In the unweighted model, age was negatively associated with L4/5 height index change (β = −0.150 per year, 95% CI −0.284 to −0.015, *p* = 0.029), but this association was attenuated after FDR correction (q = 0.084). In the baseline-severity-adjusted model, age remained nominally associated with greater L4/5 height index loss (β = −0.169 per year, 95% CI −0.302 to −0.037, *p* = 0.013; FDR *q* = 0.051), and baseline lumbar BMD was negatively associated with change in L4/5 height index (β = −10.664, 95% CI −18.839 to −2.490, *p* = 0.011; FDR *q* = 0.049). For change in L5/S1 height index, anabolic therapy was positively associated with the outcome in the unweighted model (β = 3.803, 95% CI 1.014 to 6.592, *p* = 0.008; FDR *q* = 0.040). PI was also strongly and positively associated with change in L5/S1 height index (β = 0.246 per degree, 95% CI 0.145 to 0.346, *p* < 0.001; FDR *q* < 0.001). After adjustment for baseline lumbar BMD and baseline TLK, the anabolic therapy coefficient remained nominally significant but did not remain significant after FDR correction (β = 3.478, 95% CI 0.042 to 6.914, *p* = 0.047; FDR *q* = 0.111). PI remained robustly associated with L5/S1 height index preservation after baseline-severity adjustment (β = 0.243 per degree, 95% CI 0.140 to 0.346, *p* < 0.001; FDR *q* < 0.001). These findings suggest that PI was the most consistent predictor of lower lumbosacral morphometric change across models, whereas treatment-related associations were more sensitive to baseline-severity adjustment and multiplicity correction.

### 3.4. Propensity-Score-Weighted Sensitivity Analyses

Propensity-score models and weighted sensitivity analyses were performed to further evaluate potential confounding by indication (Supplementary Tables S1–S5). The propensity score model included age, PI, baseline lumbar BMD, and baseline TLK. Older age was associated with a higher likelihood of receiving anabolic therapy (odds ratio [OR] = 1.155 per year, *p* = 0.030), whereas higher PI was associated with a lower likelihood of anabolic therapy (OR = 0.943 per degree, *p* = 0.044). Baseline lumbar BMD was strongly inversely associated with anabolic therapy assignment, and baseline TLK was positively associated with anabolic therapy assignment (OR = 1.096 per degree, *p* = 0.005), suggesting substantial confounding by baseline disease severity. Baseline balance diagnostics showed marked unweighted imbalance in several key covariates, including age, baseline lumbar BMD, baseline L4 BMD, and TLK. Overlap weighting improved balance for the propensity-score model covariates, whereas stabilized IPTW produced extreme weights. The maximum stabilized IPTW was 35.73, and the effective sample size in the control group was 9.44, indicating limited treatment-group overlap and a potential positivity concern. Therefore, the weighted analyses were interpreted as sensitivity analyses rather than primary causal estimates.

For L5 anterior height change, the treatment association observed in the unweighted model was not supported by weighted sensitivity analyses. The IPTW weighted marginal treatment effect was essentially null (β = −0.024 mm, 95% CI −1.704 to 1.655, *p* = 0.977), and the overlap-weighted marginal effect was also nonsignificant (β = −0.833 mm, 95% CI −2.102 to 0.435, *p* = 0.196). In weighted original-covariate models, anabolic therapy was likewise not significantly associated with L5 anterior height change using either IPTW (β = −0.343 mm, *p* = 0.647) or overlap weighting (β = −0.776 mm, *p* = 0.310). For L5/S1 height index, the unweighted and baseline-severity-adjusted models suggested a positive association between anabolic therapy and height index preservation, but this association was not robust in weighted sensitivity analyses. The IPTW weighted marginal effect was nonsignificant (β = −0.317, *p* = 0.899), as was the overlap-weighted marginal effect (β = 2.508, *p* = 0.332). In weighted original-covariate models, the IPTW estimate remained nonsignificant (β = 0.488, *p* = 0.808), whereas the overlap-weighted original-covariate model showed only a nonsignificant trend (β = 3.467, *p* = 0.068; FDR *q* = 0.270).

Overall, the sensitivity analyses indicate that the unweighted association between anabolic therapy and L5 anterior vertebral height loss was attenuated after baseline-severity adjustment and was not supported by IPTW or overlap-weighted analyses. The association between anabolic therapy and L5/S1 height index preservation remained nominal in the baseline-severity-adjusted model but was not robust after FDR correction or propensity-score weighting. Accordingly, treatment-related morphometric findings should be interpreted as exploratory, treatment-associated observations rather than causal treatment effects. In contrast, PI showed a more consistent level-specific association with lower lumbar morphometric change, particularly a negative association with L5 anterior height change and a positive association with L5/S1 height index preservation.

## 4. Discussion

This 24-month retrospective longitudinal study of 128 postmenopausal women with osteoporosis demonstrated that bone-targeted therapy was accompanied by significant lumbar BMD improvement, while lower lumbar vertebral and disc morphometric changes followed distinct, level-specific patterns. In unweighted models, apparent treatment-related associations were observed for L5 anterior vertebral height loss and L5/S1 disc height index preservation. However, these associations were attenuated after baseline-severity adjustment and were not statistically robust after FDR correction or propensity-score-weighted sensitivity analyses. Therefore, the primary treatment-related findings should be interpreted as exploratory, treatment-associated observations rather than causal treatment effects.

The cautious interpretation of the anabolic therapy finding is particularly important because treatment allocation in this real-world cohort was not randomized. Patients receiving anabolic therapy had lower baseline lumbar BMD and greater baseline thoracolumbar kyphosis, suggesting more severe baseline skeletal disease and raising the possibility of confounding by indication. In the baseline-severity-adjusted model, the association between anabolic therapy and L5 anterior height change was attenuated after adjustment for baseline lumbar BMD and baseline TLK. Propensity-score sensitivity analyses provided additional support for this cautious interpretation, as the IPTW-weighted and overlap-weighted marginal estimates for L5 anterior height change were nonsignificant. These findings indicate that the unweighted association between anabolic therapy and L5 anterior height loss may partly reflect baseline disease severity rather than a direct treatment effect. A reduction in BMD often leads to vertebral microfractures and progressive thoracolumbar kyphosis, which shifts the center of gravity anteriorly and increases the moment arm on the vertebral column [17]. This deterioration in sagittal alignment further elevates the risk of subsequent fractures [18]. Consequently, integrated evaluation of BMD, baseline deformity, and spinopelvic parameters is essential for interpreting longitudinal spinal morphometric change in real-world osteoporotic populations.

Accordingly, the present data do not support a causal deleterious effect of anabolic therapy on L5 vertebral height. Although anabolic agents such as teriparatide and romosozumab can stimulate bone formation, improve trabecular microarchitecture, and alter bone remodeling dynamics [19,20], any mechanistic explanation linking anabolic therapy to cortical remodeling, microcollapse, or load redistribution in the present cohort should be considered an untested hypothesis rather than a finding demonstrated by this study. The L5 vertebra serves as the keystone of the lumbar arch, sustaining high axial compression and anterior shear stress at the lumbosacral junction [21,22]. In patients with advanced baseline osteoporosis and greater thoracolumbar kyphosis, this region may be particularly vulnerable to progressive anterior wedging or subtle height reduction under sustained loading. Nevertheless, because this study relied on plain radiography at two time points only, it cannot determine whether the observed L5 height change represents an early transient phenomenon, a persistent structural change, projectional variation, or progression of pre-existing deformity.

The apparent preservation of the L5/S1 disc height index in the anabolic group should also be interpreted cautiously. Although anabolic therapy was positively associated with L5/S1 height index change in the unweighted model and remained nominally significant after baseline-severity adjustment, this association did not remain significant after FDR correction and was not robust in IPTW or overlap-weighted sensitivity analyses. Therefore, this observation should not be considered confirmatory evidence that anabolic therapy preserves the L5/S1 disc. A possible biomechanical explanation is that changes in vertebral body geometry or load redistribution may alter disc-space loading at the lumbosacral junction [23]. However, this explanation remains hypothetical. The present study did not include MRI-based disc composition analysis, CT-based endplate assessment, or serial imaging capable of identifying the temporal sequence between vertebral height change and disc height index change. Compared with the existing literature, which has mainly focused on fracture-risk reduction and global BMD gains [24,25], the present study highlights the potential value of level-specific morphometric monitoring, but the treatment-related findings should be considered exploratory and require confirmation.

PI emerged as the most consistent biomechanical determinant of lower lumbar morphometric change. In both unweighted and baseline-severity-adjusted models, higher PI was associated with greater L5 anterior height loss and better preservation of the L5/S1 disc height index. These divergent associations suggest that a fixed spinopelvic anatomical parameter may influence how mechanical stress is distributed between the L5 vertebral body and the adjacent lumbosacral disc. As a fixed anatomical constant, a high PI is fundamentally linked to greater lumbar lordosis and a steeper sacral slope to maintain sagittal balance [26]. Specifically, the steep sacral slope associated with high PI increases the resultant shear forces parallel to the sacral endplate, which may exacerbate stress on the anterior column of the L5 vertebral body [27]. This may explain why higher PI was associated with greater L5 anterior height loss. Conversely, a hyperlordotic configuration in individuals with high PI may shift part of the axial load toward the posterior elements, thereby reducing compressive load across the L5/S1 disc space compared with a more vertically loaded low-PI configuration [27]. Previous studies have focused extensively on the relationship between PI and global sagittal balance or patient-reported outcomes [28,29]. The present findings extend this concept by suggesting that PI may also modulate level-specific vertebral and disc morphometry in osteoporotic patients.

Age showed a nominal association with L4/5 disc height index loss in the baseline-severity-adjusted model, although this finding did not clearly exceed the FDR-adjusted threshold and should therefore be interpreted cautiously. The direction of this association remains biologically plausible. Disc degeneration is a complex, multifactorial process driven primarily by cellular senescence, proteoglycan depletion, and collagenous extracellular matrix degradation [30,31]. With age, calcification and sclerosis of the vertebral endplates further impair diffusion of oxygen, glucose, and other nutrients to avascular disc cells [32,33]. These biological mechanisms may explain why disc height changes are not fully captured by BMD improvement or anti-osteoporotic treatment exposure. Bone-targeted therapies, including bisphosphonates and intermittent parathyroid hormone, primarily act on vascularized bone remodeling units. In contrast, the intervertebral disc is the largest avascular structure in the human body, which may limit the ability of systemic pharmacological agents to directly modify disc degeneration [34]. While some studies have suggested that PTH interacts with PTH1R receptors on disc cells to influence matrix markers [35], these biological effects may be insufficient to overcome the mechanical and metabolic drivers of age-related degeneration. Beyond bone-targeted strategies to improve skeletal stability, interventions directed at disc health, including physical therapy and potentially regenerative or cell-based therapies, may be more relevant for preserving disc and spinal function [36,37,38].

The propensity-score analyses provide an important methodological perspective for interpreting the observational treatment comparison. The propensity score model indicated that anabolic therapy was preferentially prescribed to patients with greater baseline disease severity, including lower baseline lumbar BMD and greater TLK. Although IPTW and overlap weighting were applied to reduce measured confounding, stabilized IPTW produced extreme weights and a markedly reduced effective sample size in the control group, indicating limited treatment-group overlap and a potential positivity concern. Therefore, the weighted estimates should be interpreted as sensitivity analyses rather than definitive causal estimates. The attenuation of the L5 anterior height association after baseline-severity adjustment and the nonsignificant IPTW and overlap-weighted estimates suggest that the unweighted treatment association is not sufficiently stable to support a causal conclusion. These findings emphasize that real-world treatment allocation and baseline skeletal severity are central to the observed morphometric differences.

### Strengths, Limitations, and Future Research Directions

The strengths of this study include the 128-patient analytic cohort, available 24-month follow-up data for the primary morphometric outcomes, comprehensive morphometric assessment of both vertebral bodies and intervertebral discs, standardized radiographic measurement protocols, and multivariable regression models incorporating clinically relevant biomechanical parameters. Another strength is the use of baseline-severity-adjusted regression and propensity-score-weighted sensitivity analyses to address confounding by indication. Reporting FDR-adjusted q-values also allowed more cautious interpretation of multiple exploratory regression coefficients.

Several limitations should be emphasized. First, the observational design with nonrandomized treatment allocation introduces substantial confounding by indication. Patients receiving anabolic therapy had lower baseline lumbar BMD and greater TLK, indicating more severe baseline disease. Although baseline-severity adjustment, IPTW, and overlap weighting were applied, residual confounding cannot be excluded. In addition, several potentially important clinical confounders were not systematically available or were not included in the adjusted models, including prior vertebral fractures, previous osteoporosis treatment, vitamin D or calcium supplementation, corticosteroid exposure, comorbidities, fall history, BMI, physical activity level, and treatment adherence. These unmeasured factors may have influenced both treatment selection and longitudinal morphometric change. Second, the anabolic subgroup remained modest in size, with only 30 patients, limiting statistical power and preventing reliable anabolic-subgroup-specific multivariable modeling. Treatment heterogeneity is another limitation. The anabolic group included both teriparatide and romosozumab, whereas the non-anabolic group included zoledronic acid, denosumab, and raloxifene. These agents have different mechanisms of action and may not be biologically interchangeable. Therefore, the present study cannot determine agent-specific morphometric effects. Third, the IPTW analysis showed extreme weights and a low effective sample size in the control group, suggesting limited overlap between treatment groups; therefore, IPTW results should be interpreted only as sensitivity analyses rather than causal estimates. Fourth, radiographic measurements are subject to positioning variability, magnification, projectional distortion, and the inherent limitations of two-dimensional imaging. Using the 20-patient test–retest reliability subsample, the MDC95 for L5 anterior height was approximately 1.7 mm, which was smaller than the 2.63 mm mean decline observed in the anabolic group. This suggests that the average L5 anterior height change likely exceeded measurement error; however, smaller morphometric changes elsewhere in the study should be interpreted with this measurement margin in mind. Fifth, because morphometric measurements were available only at baseline and 24 months, this study cannot determine whether L5 height loss occurred early, progressed continuously, or reflected a stable deformity present before treatment. Serial radiographic or advanced imaging assessments at 6, 12, and 24 months would be necessary to clarify temporal dynamics. Sixth, no CT-based cortical assessment, MRI-based disc evaluation, vertebral fracture assessment, or endplate integrity analysis was performed; therefore, proposed mechanisms involving cortical porosity, microcollapse, endplate loading, or load redistribution remain untested hypotheses. Seventh, BMD outcomes were restricted to the lumbar spine, and femoral neck or total hip BMD was not systematically available for longitudinal comparison. Lumbar BMD may also be influenced by degenerative artifacts, including osteophytes, facet arthropathy, endplate sclerosis, and vascular calcification. Eighth, No patient-reported or functional outcomes were available, and this study cannot determine whether the observed morphometric changes correlate with pain, disability, incident vertebral fractures, functional status, or quality of life. Finally, the single-center design and restriction to postmenopausal Taiwanese women may limit generalizability to men, other ethnic groups, and other healthcare systems.

Future studies should employ prospective, adequately powered, multicenter designs with balanced treatment groups or randomized allocation where feasible. Serial imaging should be incorporated to determine the temporal evolution of lower lumbar morphometric changes. Quantitative CT may clarify cortical and trabecular structural adaptation, whereas high-resolution MRI may define disc composition, endplate integrity, and inflammatory or degenerative disc changes. Future studies should also incorporate validated patient-reported and functional outcomes, including pain, disability, mobility, incident vertebral fracture, and quality-of-life measures. Such data would help determine whether level-specific morphometric changes represent clinically meaningful structural deterioration or radiographic surrogate findings.

## 5. Conclusions

In this 24-month retrospective exploratory cohort study of 128 postmenopausal women with osteoporosis, bone-targeted therapy was accompanied by significant lumbar BMD improvement, while lower lumbar vertebral and disc morphometric changes varied by spinal level and baseline biomechanical context. Apparent unweighted treatment-related associations with L5 anterior vertebral height loss and L5/S1 disc height index preservation were attenuated after adjustment for baseline lumbar BMD and baseline thoracolumbar kyphosis and were not statistically robust after FDR correction or propensity-score-weighted sensitivity analyses. Given substantial confounding by indication and limited treatment-group overlap, these treatment-related findings should be interpreted as hypothesis-generating associations rather than causal treatment effects. PI showed more consistent level-specific associations, with higher PI related to greater L5 anterior height loss but better preservation of the L5/S1 disc height index. These findings support comprehensive morphometric assessment beyond BMD monitoring and suggest that spinopelvic parameters may warrant further evaluation for risk stratification in osteoporotic patients. Because patient-reported outcomes, pain scores, disability measures, incident fracture data, and quality-of-life endpoints were unavailable, the clinical significance of these radiographic morphometric changes remains uncertain. Prospective, adequately powered, confounding-adjusted studies incorporating serial imaging and clinical outcomes are required to confirm these findings.

## Figures and Tables

**Table 1:** Baseline characteristics (*n* = 128).

Item	Total	Anabolic Yes	Anabolic No	*p*-Value	SMD
*n*	128	30	98		
Anti-osteoporotic medication				<0.001	
Aclasta	18 (14.1%)	0 (0.0%)	18 (18.4%)		
Denosumab	65 (50.8%)	0 (0.0%)	65 (66.3%)		
Evenity	5 (3.9%)	5 (16.7%)	0 (0.0%)		
Forteo	25 (19.5%)	25 (83.3%)	0 (0.0%)		
Raloxifene	15 (11.7%)	0 (0.0%)	15 (15.3%)		
Age	67.77 ± 6.41	70.53 ± 4.26	66.93 ± 6.73	<0.001	0.58
L BMD (average)	0.890 ± 0.130	0.763 ± 0.126	0.929 ± 0.104	<0.001	−1.51
L4 BMD	0.978 ± 0.172	0.792 ± 0.126	1.034 ± 0.142	<0.001	−1.75
PI	49.41 ± 11.59	45.88 ± 13.74	50.49 ± 10.69	0.099	−0.40
TLK	13.54 ± 10.23	20.02 ± 10.16	11.56 ± 9.45	<0.001	0.88
ULL	20.89 ± 9.74	19.84 ± 8.89	21.21 ± 10.01	0.475	−0.14
LLL	26.26 ± 10.42	27.28 ± 11.17	25.95 ± 10.22	0.563	0.13
SS	31.76 ± 8.70	32.73 ± 7.60	31.48 ± 9.01	0.463	0.14
L4 ant. height	28.62 ± 2.54	29.44 ± 2.44	28.37 ± 2.53	0.043	0.42
L4 post. height	26.81 ± 2.63	26.56 ± 3.03	26.88 ± 2.51	0.604	−0.12
L5 ant. height	28.44 ± 3.48	29.12 ± 1.97	28.24 ± 3.80	0.094	0.26
L5 post. height	25.04 ± 2.93	24.84 ± 2.77	25.10 ± 2.99	0.662	−0.09
L4L width	35.17 ± 3.57	35.83 ± 2.26	34.97 ± 3.87	0.133	0.24
L5U width	35.79 ± 3.54	37.23 ± 2.50	35.35 ± 3.70	0.002	0.54
L5L width	33.98 ± 3.19	34.08 ± 3.32	33.95 ± 3.17	0.848	0.04
S1 vertebral width	33.32 ± 3.83	33.32 ± 4.45	33.32 ± 3.64	0.999	−0.00
L4/5 ant. height	11.55 ± 3.42	13.29 ± 3.46	11.02 ± 3.24	0.003	0.69
L4/5 post. height	7.64 ± 2.45	8.63 ± 1.78	7.34 ± 2.56	0.003	0.54
L5/S1 ant. height	12.55 ± 5.16	14.69 ± 6.52	11.89 ± 4.50	0.035	0.55
L5/S1 post. height	7.13 ± 2.66	7.77 ± 2.71	6.93 ± 2.63	0.145	0.31
L4/5 height index	27.03 ± 7.18	30.13 ± 7.06	26.08 ± 6.98	0.008	0.58
L5/S1 height index	29.51 ± 10.75	33.93 ± 12.67	28.16 ± 9.77	0.027	0.55

Continuous variables are shown as mean ± SD. Between-group *p*-values for continuous variables were calculated with Welch’s *t*-test. Abbreviations: SMD, standardized mean difference; BMD, bone mineral density; PI, pelvic incidence; TLK, thoracolumbar kyphosis; ULL, upper lumbar lordosis; LLL, lower lumbar lordosis; SS, sacral slope.

**Table 2:** Changes from baseline to 24-month follow-up (*n* = 128).

Item	Anabolic Agent	*n*	Baseline	24-Month Follow-Up	Diff. Value	*p*-Value Within Group	*p*-Value Between Group
L BMD (average)	No	98	0.929 ± 0.104	0.960 ± 0.110	0.031 ± 0.070	<0.001	0.055
	Yes	30	0.763 ± 0.126	0.826 ± 0.069	0.062 ± 0.078	<0.001	
L4 BMD	No	98	1.034 ± 0.142	1.083 ± 0.140	0.048 ± 0.072	<0.001	0.073
	Yes	30	0.792 ± 0.126	0.878 ± 0.077	0.086 ± 0.104	<0.001	
TLK	No	98	11.56 ± 9.45	10.97 ± 9.25	−0.59 ± 3.71	0.120	0.763
	Yes	30	20.02 ± 10.16	19.75 ± 13.33	−0.27 ± 5.30	0.780	
ULL	No	98	21.21 ± 10.01	20.65 ± 9.35	−0.56 ± 4.45	0.213	0.661
	Yes	30	19.84 ± 8.89	19.86 ± 11.00	0.03 ± 6.89	0.984	
LLL	No	98	25.95 ± 10.22	24.84 ± 9.70	−1.10 ± 7.83	0.166	0.211
	Yes	30	27.28 ± 11.17	28.50 ± 7.47	1.22 ± 9.03	0.466	
SS	No	98	31.48 ± 9.01	31.03 ± 8.31	−0.44 ± 6.49	0.501	0.771
	Yes	30	32.73 ± 7.60	29.37 ± 10.99	−1.18 ± 13.31	0.034	
L4 ant. height	No	98	28.37 ± 2.53	27.54 ± 2.15	−0.83 ± 2.06	<0.001	0.634
	Yes	30	29.44 ± 2.44	28.84 ± 2.29	−0.60 ± 2.40	0.184	
L4 post. height	No	98	26.88 ± 2.51	26.31 ± 2.21	−0.57 ± 2.43	0.022	<0.001
	Yes	30	26.56 ± 3.03	27.51 ± 2.06	0.95 ± 1.52	0.002	
L5 ant. height	No	98	28.24 ± 3.80	27.90 ± 3.63	−0.34 ± 3.51	0.344	0.002
	Yes	30	29.12 ± 1.97	26.49 ± 3.68	−2.63 ± 3.27	<0.001	
L5 post. height	No	98	25.10 ± 2.99	25.32 ± 2.99	0.22 ± 2.90	0.461	0.466
	Yes	30	24.84 ± 2.77	24.79 ± 2.28	−0.05 ± 1.20	0.817	
L4L width	No	98	34.97 ± 3.87	33.99 ± 2.75	−0.98 ± 3.25	0.004	0.291
	Yes	30	35.83 ± 2.26	35.42 ± 3.01	−0.40 ± 2.36	0.356	
L5U width	No	98	35.35 ± 3.70	35.37 ± 3.17	0.01 ± 2.97	0.969	0.778
	Yes	30	37.23 ± 2.50	36.99 ± 4.51	−0.24 ± 4.59	0.776	
L5L width	No	98	33.95 ± 3.17	34.03 ± 3.53	0.08 ± 3.25	0.813	0.461
	Yes	30	34.08 ± 3.32	34.60 ± 2.43	0.52 ± 2.76	0.307	
S1 vertebral width	No	98	33.32 ± 3.64	32.65 ± 3.49	−0.68 ± 3.65	0.070	0.808
	Yes	30	33.32 ± 4.45	32.41 ± 4.91	−0.91 ± 4.93	0.319	
L4/5 ant. height	No	98	11.02 ± 3.24	10.77 ± 3.77	−0.25 ± 2.58	0.348	0.088
	Yes	30	13.29 ± 3.46	14.08 ± 3.30	0.80 ± 2.95	0.149	
L4/5 post. height	No	98	7.34 ± 2.56	6.76 ± 2.05	−0.58 ± 1.89	0.003	0.386
	Yes	30	8.63 ± 1.78	7.72 ± 0.90	−0.91 ± 1.82	0.010	
L5/S1 ant. height	No	98	11.89 ± 4.50	11.28 ± 4.53	−0.62 ± 2.18	0.006	0.119
	Yes	30	14.69 ± 6.52	15.49 ± 5.40	0.81 ± 4.73	0.356	
L5/S1 post. height	No	98	6.93 ± 2.63	6.82 ± 2.60	−0.12 ± 1.71	0.495	0.100
	Yes	30	7.77 ± 2.71	8.15 ± 3.34	0.38 ± 1.34	0.130	
L4/5 height index	No	98	26.08 ± 6.98	25.21 ± 7.09	−0.87 ± 4.65	0.066	0.321
	Yes	30	30.13 ± 7.06	30.22 ± 4.49	0.10 ± 4.65	0.908	
L5/S1 height index	No	98	28.16 ± 9.77	27.24 ± 9.97	−0.93 ± 5.37	0.091	0.109
	Yes	30	33.93 ± 12.67	36.26 ± 15.21	2.33 ± 10.43	0.230	

Within-group *p*-values were calculated with paired *t*-tests; between-group *p*-values compare diff. values using Welch’s *t*-test. Diff. value = 24-month follow-up value minus baseline value; negative values indicate height/index loss or angle decrease. Abbreviations: BMD, bone mineral density; TLK, thoracolumbar kyphosis; ULL, upper lumbar lordosis; LLL, lower lumbar lordosis; SS, sacral slope.

**Table 3:** Unweighted and baseline-severity-adjusted regression models.

Outcome	Model	Predictor	β	95% CI Lower	95% CI Upper	*p*-Value	FDR *q*-Value	Adjusted R^2^	*n*	*n*/Predictors
Diff. of L4 ant. height	Model 1: unweighted	Age	0.058	−0.003	0.12	0.064	0.128	0.037	128	32
		Anabolic agent (Yes vs. No)	−0.209	−1.142	0.724	0.659	0.795	0.037	128	32
		PI	−0.008	−0.042	0.025	0.626	0.783	0.037	128	32
		Change in L BMD	6.182	0.976	11.388	0.020	0.067	0.037	128	32
	Model 2: baseline-severity-adjusted	Age	0.07	0.01	0.129	0.022	0.067	0.116	128	21.3
		Anabolic agent (Yes vs. No)	0.829	−0.264	1.922	0.136	0.247	0.116	128	21.3
		PI	−0.004	−0.036	0.029	0.822	0.914	0.116	128	21.3
		Change in L BMD	10.561	4.963	16.159	<0.001	0.002	0.116	128	21.3
		Baseline L BMD	6.645	2.981	10.308	<0.001	0.003	0.116	128	21.3
		Baseline TLK	−0.011	−0.049	0.026	0.551	0.766	0.116	128	21.3
Diff. of L5 ant. height	Model 1: unweighted	Age	0.09	−0.005	0.185	0.062	0.128	0.187	128	32
		Anabolic agent (Yes vs. No)	−2.795	−4.228	−1.363	<0.001	0.001	0.187	128	32
		PI	−0.105	−0.156	−0.053	<0.001	0.001	0.187	128	32
		Change in L BMD	−9.885	−17.878	−1.892	0.016	0.057	0.187	128	32
	Model 2: baseline-severity-adjusted	Age	0.1	0.006	0.194	0.037	0.094	0.209	128	21.3
		Anabolic agent (Yes vs. No)	−1.64	−3.368	0.088	0.063	0.128	0.209	128	21.3
		PI	−0.104	−0.155	−0.052	<0.001	0.001	0.209	128	21.3
		Change in L BMD	−6.185	−15.036	2.666	0.169	0.294	0.209	128	21.3
		Baseline L BMD	6.157	0.365	11.949	0.037	0.094	0.209	128	21.3
		Baseline TLK	−0.033	−0.093	0.026	0.269	0.399	0.209	128	21.3
Diff. of L4/5 height index	Model 1: unweighted	Age	−0.15	−0.284	−0.015	0.029	0.084	0.031	128	32
		Anabolic agent (Yes vs. No)	1.37	−0.663	3.403	0.185	0.308	0.031	128	32
		PI	0.02	−0.053	0.093	0.588	0.766	0.031	128	32
		Change in L BMD	7.475	−3.87	18.82	0.195	0.311	0.031	128	32
	Model 2: baseline-severity-adjusted	Age	−0.169	−0.302	−0.037	0.013	0.051	0.067	128	21.3
		Anabolic agent (Yes vs. No)	−0.074	−2.513	2.365	0.952	0.993	0.067	128	21.3
		PI	0.009	−0.064	0.082	0.801	0.914	0.067	128	21.3
		Change in L BMD	0.04	−12.451	12.531	0.995	0.995	0.067	128	21.3
		Baseline L BMD	−10.664	−18.839	−2.49	0.011	0.049	0.067	128	21.3
		Baseline TLK	−0.008	−0.092	0.076	0.856	0.925	0.067	128	21.3
Diff. of L5/S1 height index	Model 1: unweighted	Age	0.055	−0.129	0.239	0.555	0.766	0.192	128	32
		Anabolic agent (Yes vs. No)	3.803	1.014	6.592	0.008	0.040	0.192	128	32
		PI	0.246	0.145	0.346	<0.001	<0.001	0.192	128	32
		Change in L BMD	12.527	−3.036	28.09	0.114	0.217	0.192	128	32
	Model 2: baseline-severity-adjusted	Age	0.05	−0.136	0.237	0.594	0.766	0.179	128	21.3
		Anabolic agent (Yes vs. No)	3.478	0.042	6.914	0.047	0.111	0.179	128	21.3
		PI	0.243	0.14	0.346	<0.001	<0.001	0.179	128	21.3
		Change in L BMD	10.817	−6.78	28.415	0.226	0.348	0.179	128	21.3
		Baseline L BMD	−2.438	−13.954	9.078	0.676	0.795	0.179	128	21.3
		Baseline TLK	−0.002	−0.121	0.116	0.968	0.993	0.179	128	21.3

Model 1 predictors: age, anabolic agent, PI, and change in lumbar BMD. Model 2 additionally adjusts for baseline lumbar BMD and baseline TLK. FDR *q-*values were calculated by Benjamini–Hochberg correction across the non-intercept coefficients in the main Table 3 models. Abbreviations: CI, confidence interval; PI, pelvic incidence; BMD, bone mineral density; TLK, thoracolumbar kyphosis.

## Data Availability

Data are contained within the article.

## Supplementary Materials

The following supporting information can be downloaded at: https://www.mdpi.com/article/10.3390/bioengineering13070800/s1, Table S1: IPTW and overlap-weighted treatment-effect sensitivity analyses; Table S2: Full IPTW and overlap-weighted model coefficients; Table S3: Propensity score logistic regression model; Table S4. Propensity-score weight diagnostics; Table S5: Baseline balance before and after weighting.

## Abbreviations

BMD	Bone mineral density
BMI	Body mass index
DHI	Disc Height Index
Di	Inferior Disk Depth
Ds	Superior Disk Depth
DXA	Dual-energy X-ray absorptiometry
GT	Global Tilt
ICC	Intraclass correlation coefficient
LL	Lumbar lordosis/total lumbar lordosis
LLL	Lower lumbar lordosis
SS	Sacral slope
PACS	Picture Archiving and Communication System
SD	Standard deviation
TLK	Thoracolumbar kyphosis
ULL	Upper lumbar lordosis
