# Supplementary material for: Bone-Targeted Therapies and Spinal Morphometry in Postmenopausal Osteoporosis: A Real-World Longitudinal Retrospective Study
Source: Bioengineering (Basel). 2026 Jul 13;13(7):800. doi: 10.3390/bioengineering13070800 (PMC13405480; doi:10.3390/bioengineering13070800)
Supplement: Supplementary file 1 [file bioengineering-13-00800-s001.zip › bioengineering-4359085-supplementary.pdf]

Table S1. IPTW and overlap-weighted treatment-effect sensitivity analyses.

Outcome	Model	Treatment β (Anabolic Yes vs No)	95% CI lower	95% CI upper	*p*-value	FDR q-value	Adjusted R^2	*n*	n/predictors
Diff. of L4 ant. height	IPTW weighted marginal	1.356	0.42	2.292	0.005	0.048	0.046	128	128
	Overlap-weighted marginal	0.832	-0.312	1.976	0.153	0.339	0.028	128	128
	IPTW weighted original-covariate model	1.342	0.337	2.346	0.009	0.074	0.092	128	32
	Overlap-weighted original-covariate model	1.016	-0.147	2.179	0.086	0.270	0.097	128	32
Diff. of L5 ant. height	IPTW weighted marginal	-0.024	-1.704	1.655	0.977	0.977	-0.008	128	128
	Overlap-weighted marginal	-0.833	-2.102	0.435	0.196	0.386	0.016	128	128
	IPTW weighted original-covariate model	-0.343	-1.823	1.137	0.647	0.822	0.071	128	32
	Overlap-weighted original-covariate model	-0.776	-2.282	0.73	0.310	0.476	0.026	128	32
Diff. of L4/5 height index	IPTW weighted marginal	-1.894	-4.186	0.398	0.104	0.279	0.011	128	128
	Overlap-weighted marginal	-0.986	-3.136	1.163	0.365	0.522	0.007	128	128
	IPTW weighted original-covariate model	-2.006	-4.027	0.014	0.052	0.258	0.065	128	32
	Overlap-weighted original-covariate model	-0.999	-3.362	1.363	0.404	0.557	0.051	128	32
Diff. of L5/S1 height index	IPTW weighted marginal	-0.317	-5.251	4.616	0.899	0.946	-0.008	128	128
	Overlap-weighted marginal	2.508	-2.593	7.608	0.332	0.492	0.014	128	128
	IPTW weighted original-covariate model	0.488	-3.473	4.45	0.808	0.897	0.114	128	32
	Overlap-weighted original-covariate model	3.467	-0.264	7.197	0.068	0.270	0.406	128	32

Overlap weights were calculated as 1 - PS for treated participants and PS for control participants. Weighted models use HC3 robust standard errors. Because stabilized IPTW contained extreme weights, these models should be reported as sensitivity analyses. Abbreviations: IPTW, stabilized inverse probability of treatment weighting; CI, confidence interval.

 https://doi.org/10.3390/bioengineering13070800

Table S2. Full IPTW and overlap-weighted model coefficients.

Outcome	Model	Predictor	β	95% CI lower	95% CI upper	*p*-value	FDR *q*-value	Adjusted R^2	*n*	*n*/predictors
Diff. of L4 ant. height	IPTW weighted marginal	Anabolic agent (Yes vs No)	1.356	0.42	2.292	0.005	0.048	0.046	128	128
	Overlap-weighted marginal	Anabolic agent (Yes vs No)	0.832	-0.312	1.976	0.153	0.339	0.028	128	128
	IPTW weighted original-covariate model	Age	-0.005	-0.06	0.05	0.863	0.933	0.092	128	32
		Anabolic agent (Yes vs No)	1.342	0.337	2.346	0.009	0.074	0.092	128	32
		PI	0.018	-0.014	0.05	0.262	0.420	0.092	128	32
		Change in L BMD	7.056	1.384	12.727	0.015	0.101	0.092	128	32
	Overlap-weighted original-covariate model	Age	0.106	-0.026	0.239	0.114	0.286	0.097	128	32
		Anabolic agent (Yes vs No)	1.016	-0.147	2.179	0.086	0.270	0.097	128	32
		PI	0.032	-0.02	0.083	0.229	0.403	0.097	128	32
		Change in L BMD	5.978	-1.162	13.118	0.100	0.279	0.097	128	32
Diff. of L5 ant. height	IPTW weighted marginal	Anabolic agent (Yes vs No)	-0.024	-1.704	1.655	0.977	0.977	-0.008	128	128
	Overlap-weighted marginal	Anabolic agent (Yes vs No)	-0.833	-2.102	0.435	0.196	0.386	0.016	128	128
	IPTW weighted original-covariate model	Age	-0.126	-0.292	0.039	0.132	0.310	0.071	128	32
		Anabolic agent (Yes vs No)	-0.343	-1.823	1.137	0.647	0.822	0.071	128	32
		PI	-0.056	-0.119	0.007	0.083	0.270	0.071	128	32
		Change in L BMD	-4.932	-13.238	3.374	0.242	0.403	0.071	128	32
	Overlap-weighted original-covariate model	Age	0.085	-0.167	0.337	0.505	0.673	0.026	128	32
		Anabolic agent (Yes vs No)	-0.776	-2.282	0.73	0.310	0.476	0.026	128	32
		PI	-0.044	-0.107	0.019	0.167	0.352	0.026	128	32
		Change in L BMD	1.846	-8.861	12.554	0.733	0.873	0.026	128	32
Diff. of L4/5 height index	IPTW weighted marginal	Anabolic agent (Yes vs No)	-1.894	-4.186	0.398	0.104	0.279	0.011	128	128
	Overlap-weighted marginal	Anabolic agent (Yes vs No)	-0.986	-3.136	1.163	0.365	0.522	0.007	128	128
	IPTW weighted original-covariate model	Age	0.125	-0.068	0.319	0.203	0.386	0.065	128	32
		Anabolic agent (Yes vs No)	-2.006	-4.027	0.014	0.052	0.258	0.065	128	32
		PI	-0.082	-0.163	-0.001	0.046	0.258	0.065	128	32
		Change in L BMD	1.65	-8.268	11.569	0.742	0.873	0.065	128	32
	Overlap-weighted	Age	-0.229	-0.614	0.155	0.240	0.403	0.051	128	32
		Anabolic agent (Yes vs No)	-0.999	-3.362	1.363	0.404	0.557	0.051	128	32

	original-covariate model	PI	0.017	-0.096	0.131	0.763	0.873	0.051	128	32
		Change in L BMD	-0.412	-15.1	14.276	0.956	0.977	0.051	128	32
Diff. of L5/S1 height index	IPTW weighted marginal	Anabolic agent (Yes vs No)	-0.317	-5.251	4.616	0.899	0.946	-0.008	128	128
	Overlap-weighted marginal	Anabolic agent (Yes vs No)	2.508	-2.593	7.608	0.332	0.492	0.014	128	128
	IPTW weighted original-covariate model	Age	0.123	0.044	0.202	0.002	0.032	0.114	128	32
		Anabolic agent (Yes vs No)	0.488	-3.473	4.45	0.808	0.897	0.114	128	32
		PI	0.2	0.085	0.315	<0.001	0.016	0.114	128	32
		Change in L BMD	3.994	-13.799	21.787	0.658	0.822	0.114	128	32
	Overlap-weighted original-covariate model	Age	0.244	-0.035	0.524	0.086	0.270	0.406	128	32
		Anabolic agent (Yes vs No)	3.467	-0.264	7.197	0.068	0.270	0.406	128	32
		PI	0.448	0.265	0.631	<0.001	<0.001	0.406	128	32
		Change in L BMD	31.061	-4.677	66.798	0.088	0.270	0.406	128	32

Abbreviations: IPTW, stabilized inverse probability of treatment weighting; CI, confidence interval; PI, pelvic incidence; BMD, bone mineral density.

Table S3. Propensity score logistic regression model.

Predictor	Logit coefficient	95% CI lower	95% CI upper	*p*-value	Odds ratio	OR 95% CI lower	OR 95% CI upper
Intercept	4.413	-5.111	13.937	0.364	82.527	0.006	1129338.69
Age	0.144	0.014	0.274	0.030	1.155	1.014	1.315
PI	-0.059	-0.117	-0.002	0.044	0.943	0.89	0.998
Baseline lumbar BMD, per 0.1 g/cm^2 increase	-1.666	-2.444	-0.888	< 0.001	0.189	0.087	0.412
Baseline TLK	0.091	0.027	0.155	0.005	1.096	1.028	1.168

Odds ratios for baseline lumbar BMD are expressed per 0.1 g/cm^2 increase to improve interpretability. Abbreviations: CI, confidence interval; PI, pelvic incidence; BMD, bone mineral density; TLK, thoracolumbar kyphosis.

Table S4. Propensity-score weight diagnostics.

Weight	Group	*n*	Min	P1	P25	Median	P75	P99	Max	Mean	SD	Effective sample size
Unstabilized IPTW	Overall	128	1	1.001	1.011	1.092	1.366	35.811	46.663	2.124	5.732	15.562
	Anabolic Yes	30	1.006	1.006	1.285	1.489	2.274	6.47	6.47	2.178	1.767	18.335
	Anabolic No	98	1	1	1.009	1.028	1.153	46.663	46.663	2.108	6.487	9.444
Stabilized IPTW	Overall	128	0.236	0.236	0.766	0.778	0.857	27.066	35.726	1.355	4.37	11.307
	Anabolic Yes	30	0.236	0.236	0.301	0.349	0.533	1.516	1.516	0.51	0.414	18.335
	Anabolic No	98	0.766	0.766	0.772	0.787	0.883	35.726	35.726	1.614	4.966	9.444

https://doi.org/10.3390/bioengineering13070800

Overlap weight	Overall	128	0	0.001	0.01	0.084	0.268	0.943	0.979	0.171	0.232	45.487
	Anabolic Yes	30	0.006	0.006	0.221	0.328	0.538	0.845	0.845	0.366	0.251	20.607
	Anabolic No	98	0	0	0.009	0.028	0.133	0.979	0.979	0.112	0.19	25.374

Key diagnostic warning: stabilized IPTW maximum = 35.73; control-group ESS = 9.44. This suggests limited treatment-group overlap / positivity concern. Abbreviations: IPTW, stabilized inverse probability of treatment weighting; SD, Standard deviation.

Table S5. Baseline balance before and after weighting.

Covariate	Unweighted mean Yes	Unweighted mean No	Unweighted SMD	IPTW mean Yes	IPTW mean No	IPTW SMD	Overlap mean Yes	Overlap mean No	Overlap SMD
Age	70.53	66.93	0.58	70.02	71.44	-0.26	70.17	70.17	0.00
PI	45.88	50.49	-0.40	44.37	47.72	-0.31	46.36	46.36	0.00
Baseline L BMD	0.76	0.93	-1.51	0.81	0.87	-0.58	0.82	0.82	0.00
Baseline L4 BMD	0.79	1.03	-1.75	0.85	0.93	-0.63	0.85	0.92	-0.48
Baseline TLK	20.02	11.56	0.88	17.53	29.89	-0.73	18.62	18.62	0.00
Baseline ULL	19.84	21.21	-0.14	19.35	18.36	0.13	20.34	19.33	0.11
Baseline LLL	27.28	25.95	0.13	29.03	36.84	-0.63	28.78	29.87	-0.09
Baseline SS	32.73	31.48	0.14	36.10	32.70	0.48	36.48	32.02	0.51

Abbreviations: IPTW, stabilized inverse probability of treatment weighting; PI, pelvic incidence; BMD, bone mineral density; TLK, thoracolumbar kyphosis; ULL, upper lumbar lordosis; LLL, lower lumbar lordosis; SS, sacral slope.
